# Knowledge, perception, and attitude toward using collagen among people in the western region of Saudi Arabia: A cross-sectional study

**DOI:** 10.1097/MD.0000000000048742

**Published:** 2026-05-15

**Authors:** Ahmed M. Ashour, Saad M. Wali, Nasser M. Alorfi, Fahad S. Alshehri, Bader A. Alhazmi, Mohammed M. Aldurdunji, Beisan A. Mohammad, Mashael A. Alamri, Naif H. Ashri, Aliah Alhayyan, Ohood K. Almuzaini, Nasser M. Aldekhail

**Affiliations:** a Department of Pharmacology and Toxicology, College of Pharmacy, Umm Al-Qura University, Makkah, Saudi Arabia; b King Salman Center for Disability Research, Riyadh, Saudi Arabia; c Pharmaceutical Practices Department, College of Pharmacy, Umm Al-Qura University, Makkah, Saudi Arabia; d Pharmaceutical Sciences Department, PharmD Program, Fakeeh College for Medical Sciences, Fakeeh Care Group, Jeddah, Saudi Arabia; e Department of Biology, Faculty of Sciences, Umm Al-Qura University, Makkah, Saudi Arabia; f Third Riyadh Health Cluster, Huraymala General Hospital, Riyadh, Saudi Arabia; g Department of Pharmacy, College of Pharmacy, Nursing and Medical Sciences, Riyadh Elm University, Riyadh, Saudi Arabia.

**Keywords:** awareness, collagen, cosmetic, knowledge, western

## Abstract

Collagens are protein particles consisting of amino acids that deliver structural support to connective tissues. Collagen’s stiffness, rigidity, and resistance to stretching makes it an ideal matrix for skin, tendons, bones, and ligaments. Collagen deficiency disrupts tissue strength and function, increasing pain, mobility limitations, and disability risk. Many studies have shown that collagen supplements are safe cosmetics that can elevate collagen-derived peptides in the blood, improving skin elasticity, hair growth, wound healing, and potentially supporting weight management. However, no cross-sectional study has assessed public knowledge, perceptions, and attitudes toward collagen use in Saudi Arabia. This study aimed to evaluate these factors among adults in the western region of Saudi Arabia. A cross-sectional, web-based online survey of people living in the western region of Saudi Arabia was conducted. Data were collected between January to March 2025. As there were no immediate data regarding the total number of people using collagen in Saudi Arabia, no sampling frame was produced. Instead, a convenience sampling model was used to select the participants. All guidelines outlined in the Declaration of Helsinki were followed by the research group. A total of 368 respondents were included most of the participants were 18 to 29 years old (61.7%); 53% were female; 47% were male; 56.8% were single; 84.5% had a university-level education or higher; most were unemployed or retired (54.9%), and most earned <5000 Saudi riyals in monthly income (63%). Only 32.6% of participants reported prior collagen use, and knowledge was generally poor, particularly among younger adults and males. Collagen users demonstrated significantly higher knowledge compared to nonusers (65.8% vs 28.2%, *P* < .001). Awareness of side effects was low, with 78% of participants unaware of minor adverse effects. Public knowledge of collagen use in the western region of Saudi Arabia is limited, which may lead to misuse or unrealistic expectations. These findings underscore the need for educational interventions to promote safe and informed use of collagen products.

## 1. Introduction

### 1.1. Definitions and types

Collagens are protein particles that consist of amino acids, and they deliver structural support to the extracellular matrix of connective tissues. Due to its stiffness, rigidity, and resistance to stretching, collagen is the ideal matrix for skin, tendons, bones, and ligaments. Collagens are categorized into groups depending on the structure form, with 28 types of collagens having been identified, the most common types of which are I to IV, and in which type I includes over 90% of the collagen located in the human body. Type I collagen makes up around 95% of the total collagen content of bone and around 80% of the total protein content of bone (Table 1).^[3,4]^

**Table 1:** Types of collagen and their locations in the body and its extraneous sources.^[1,2]^

Type	Location	Sources	Characteristics
Type I	Dermis, tendon, ligaments, and bone.	Bone broth, egg whites, bovine collagen peptides, protein-rich food, and fish/marine collagen.	Bundles of fibers (2–10 µm thick). Closely packed, thick, non-argyrophilic, strongly birefringent, yellow or red fibers. Collagen fibers: densely packed, thick (75 nm) fibrils with marked variation in diameter.
Type II	Cartilage, vitreous body, and nucleus pulposus.	Bone broth, chicken collagen, protein rich foods (e.g. chicken), and multi-collagen protein powder.	Fibrils (20–30 nm thick). Loose collagenous network displaying weak birefringence of a variable color. No fibers, very thin fibrils.
Type III	Skin, vessel walls, reticular fibers of most tissues.	Bone broth, egg whites, bovine collagen peptides, protein-rich food, and collagen protein powder.	Individual fibers (0.5–1.5 µm thick). Loose network of thin, argyrophilic, weakly birefringent, greenish fibers. Reticulin fibers: loosely packed, thin. Fibrils with more uniform diameter (45 nm).
Type IV	Forms of basal lamina, the epithelium-secreted layer of the basement membranes.	Egg whites, protein-rich food. Difficult to find in supplement form.	Microfibrils containing ordered molecules (1.5 nm wide). Thin, amorphous, weakly birefringent basal laminae.

As in biochemical passageways, several steps must be carefully carried out in processing collagen, and they are tightly standardized and controlled. However, with these many steps, failures can occur due to genetic mutations, which ultimately lead to errors. These errors take place in assembly, posttranslational modification, and nutritional insufficiencies, which influence the enzymatic function of the collagen synthesized.^[5]^ Collagen synthesis is a complex, multi-step process that is susceptible to nutritional and genetic influences.^[6]^

### 1.2. Mutation and disorders of collagen

Mutations are rare, but errors of collagen synthesis can result in conditions like scurvy, osteogenesis imperfect, and Ehlers–Danlos syndrome (EDS). Ageing, autoimmune diseases, and stress can change the integrity and quantity of collagen in the skin. Collagen-related diseases occur due to genetic defects or nutritional deficiencies that affect the processes involved in normal collagen production.^[7–11]^ Important to note, certain conditions can substantially influence presence and functionality of collage. Notable among these include intake of too much sugar, excess exposure to sunlight, aging, and tobacco usage. Excess consumption of sugar may lead to negative effects on the body that damage the collagen into an unstable type I, which is weakened and can be easily broken down and lead to glycation that produces advanced glycation end-product.^[12]^ Exposure to excessive sunlight and ultraviolet (UV) radiation may decrease collagen production, while the use of tobacco can damage the skin’s collagen and alters the balance of extracellular matrix (ECM) turnover in skin.^[13–15]^ The amount of collagen in the skin changes with age, with the capacity to replenish collagen naturally reducing as can be noted in recent study pointing that collagen loss begins at 18 to 29 years of age, and by the age of 40, about 1% of collagen can be lost yearly.^[16]^ Further, it is important to point that collagen is the main component of ECM, becomes fragmented, and it decreases in collagen amount by increasing the activity of matrix metalloproteinases and impaired transforming growth factor-β signaling due to the reactive oxygen species which hinders the mechanical interaction between the ECM and fibroblasts, leading to worsened fibroblast function and a further reduction in the amount of collagen.^[17]^

#### 1.2.1. Role of collagen deficiency in disability

Collagen deficiency is increasingly recognized as a contributor not only to cosmetic and structural changes but also to functional impairment and disability.^[18]^ Because collagen is the primary structural protein in connective tissues, essentially including bone, cartilage, tendons, ligaments, skin, and the vascular system, it is important to point out here that its reduction or abnormal synthesis can substantially have far reaching effects, particularly that it can compromise the mechanical stability and functional integrity of multiple organ systems.^[19]^ Primarily, impaired collagen structure has the potential to lead to joint laxity, chronic musculoskeletal pain, reduced mobility, delayed wound healing, fragile skin, as well as a higher susceptibility to injuries, notably all of which can significantly affect an individual’s ability to perform daily activities.^[20,21]^

In hereditary disorders, particularly the likes of osteogenesis imperfecta and various forms of Ehlers–Danlos syndrome, collagen insufficiency or malformation causes frequent fractures, joint dislocations, chronic fatigue, and impaired proprioception, often resulting in long-term physical disability and reduced quality of life.^[22]^ In the same way, secondary collagen loss due to aging, nutritional deficiencies, smoking, or excessive exposure to UV light contributes to sarcopenia, decreased muscle strength, poor balance, and increased risk of falls, fundamentally occurrences that are key predictors of disability in older adults.^[23]^ Recent research further points that from a public health perspective, collagen-related deficits across the musculoskeletal and integumentary systems plays an essential role in functional decline, particularly among populations with chronic illnesses, limited mobility, or high rates of vitamin C deficiency.^[24,25]^ Accordingly, understanding collagen’s broader physiological role points to the critical importance of public awareness, early prevention, and proper management, notably as misconceptions or inadequate knowledge about collagen may delay appropriate nutritional, dermatological, or rehabilitative interventions that could mitigate disability risk.

### 1.3. Uses of collagen as a cosmetic

Collagen has dermatological properties that significantly improve skin after 1 to 3 months of consumption. In addition, it improves the characteristics of nails after 24 weeks.^[26–29]^ Collagen has also been identified as a safe cosmetic that elevates the levels of collagen-derived peptides in the blood flow and increases skin properties such as elasticity, skin moisture, and skin firmness. In addition, it may decrease the progression of skin aging and provide skin protection against UV melasma while also reducing UV spots on the skin.^[30,31]^ Other important areas of collagen significance include use of collagen as a skin enhancement, in wound healing, air care, and in weight management.^[21,32]^ A supporting element for this wide importance of and applicability of collagen is that in research, it has been identified as a safe with minimal side effects, such as nausea, flatulence, or dyspepsia, making it insignificantly harmful in the various ways it is utilized.^[1,2,33–36]^

### 1.4. Knowledge about collagen use worldwide and in Saudi Arabia

Collagen is used not only in the cosmetic industry but also in pharmaceuticals and the beverage, food, and health care sectors, driving the growth of collagen’s use worldwide. The current global market is estimated to be 3.7 billion USD and is estimated to grow to over 6.6 billion by 2025.^[37]^

In Saudi Arabia, a clinical trial was conducted in the King Saud Medical City medical unit, surgical unit and intensive care unit to compare adaptive response to collagen wound dressing in pressure ulcer treatment for 3 weeks. The results promote the use of collagen dressing in the treatment of pressure ulcers, with the need for further studies to investigate the most appropriate and cost-effective use for wound care products for the management of pressure ulcer treatment.^[31]^ Another study investigated the characteristics of acid-extracted collagen from buffalo skin using amino acid analysis methods, with the results indicating that collagen extracted from buffalo skin is a potential alternative source to pig collagen for diet, cosmetic, biomedical, and pharmaceutical products.^[38]^ A pilot study assessed the use of an acellular collagen matrix as a new method of penile augmentation. Another study found oral collagen use is popular for hair, skin, and joints, with modest benefits reported (especially by younger users after 4–8 weeks) but also common side effects like GI issues.^[39–42]^ The results demonstrate that acellular collagen matrix is unsuitable in penile girth augmentation due to reported adverse reactions, including penile edema with ischemic dorsal ulcers, bruising, and inflammation reactions. The severity of complications led the researchers to stop using this technique.^[32]^

However, no cross-sectional study has been done to assess the knowledge, perceptions, and attitudes towards the use of collagen among people in the western region of Saudi Arabia. Most notably, despite the growing popularity of collagen supplements in Saudi Arabia as indicated by the recent research in the area, public understanding of their proper use and expected outcomes remains limited. This gap in knowledge has the potential to contribute to misuse, inappropriate self-medication, or even unrealistic expectations regarding cosmetic or health benefits. Notably, no prior study has specifically examined public awareness and attitudes toward collagen use in the Western region of Saudi Arabia. Addressing this gap is therefore both timely and essential to guide safe, informed, and evidence-based use of collagen products. This study offers a novel contribution by providing the first regional assessment of these perceptions. In particular, the fundamental aim in this study was to evaluate people’s knowledge, perceptions, and attitudes towards using collagen in the western region of Saudi Arabia. We hypothesized that the general public in the western region of Saudi Arabia has limited knowledge about collagen use and that attitudes toward collagen consumption are influenced by demographic factors such as age, gender, and cosmetic use habits.

## 2. Methods

### 2.1. Study design and target population

A cross-sectional web-based survey of people living in the western region of Saudi Arabia was conducted. Data were collected between January to March 2025. As there were no available data regarding the total number of people using collagen in the western region, no sampling frame was produced as the researchers thought that such a model may limit representativeness and introduce selection bias. Instead, a convenience sampling model was used to select the participants. The participants were enrolled into the survey online. The study population included both female and male volunteers 18 years and older who were cosmetics users, particularly including both current and potential users of collagen or other cosmetic products, as the study aimed to assess general public knowledge even among nonusers. The exclusion criteria were adolescents and children younger than 18 years, people not living in the western region of Saudi Arabia, individuals with recent surgeries, and pregnant women. These revised criteria ensured consistency with the data, as individuals who had never used collagen were intentionally included to assess population-level knowledge and attitudes.

A questionnaire was used as a data collection instrument by the researchers. The document was made up of 5 sections: section I (socio-demographics), section II (participants’ knowledge), section III (factors and causes of deficiency), section IV (effectiveness of collagen), and section V (side effects). The questionnaire was critically appraised by independent experts in this field of study to determine its face validity, with 3 specialists in dermatology, nutrition, and public health reviewing item clarity and relevance. A pilot test involving 30 participants from the target population was also conducted, and internal consistency was assessed using Cronbach alpha, which ranged from 0.72 to 0.84 across the major sections, indicating acceptable reliability. Data from the pilot were excluded from the final analysis.

### 2.2. Statistical approach

All data were analyzed using Statistical Package for the Social Sciences (SPSS) version 22. This analysis was performed after a check for completeness and accuracy of the data collected from the participants. The demographic characteristics of the respondents were generated using the software’s descriptive statistic command. Frequency and percentage were used to characterize the knowledge of, attitudes towards, and perceptions of the participants in relation to collagen use. Knowledge questions were scored using a binary system (1 = Yes, 0 = No). A cutoff score of ≥60% was used to classify participants as having good knowledge, based on thresholds commonly applied in similar public awareness and health literacy studies, with scores below 60% indicating poor knowledge. No adjustments for confounding variables were performed due to the descriptive nature of the study, and the researchers fully acknowledge this aspect as as a methodological limitation.

### 2.3. Data analysis

After data were extracted, they were revised, coded, and fed to statistical software IBM SPSS version 22 (SPSS, Inc. Chicago, IL). All statistical analysis was done using two-tailed tests. *P* values of <.05 were considered statistically significant. For knowledge and awareness items, each correct answer was scored with 1 point, and the total summation of discrete scores for the different items in each domain of knowledge was calculated. A patient with a score of <60% of the total was considered to have a poor knowledge level, while a good knowledge level was indicated by 60% or more of the total score. Descriptive analyses based on frequency and percent distribution were done for all variables, including participants’ demographic data, collagen use patterns, and monthly income. In addition, participants’ knowledge and perceptions regarding collagen use were tabulated and graphed by domain. Cross-tabulation was used to assess the distribution of participants’ knowledge levels regarding collagen according to their personal data and history of use. Relations were tested using the Pearson Chi-square test and exact probability test for small frequency distributions.

### 2.4. Ethical approval

A letter of ethical approval was obtained from the ethical committee at Umm AlQura University, Saudi Arabia Approval No. (HAPO-02-K-012-2021-12-900). All guidelines outlined in the Declaration of Helsinki were followed by the research group. The online questionnaire was prepared with a preface that outlined the nature and purpose of the study and distributed via an online platform. In addition, the preface included a consent section that guaranteed anonymity and voluntary participation of the respondents.

## 3. Results

### 3.1. Demographic characteristics

A total of 368 respondents were included in the final analysis. The majority of participants were between 18 and 29 years (61.7%); 53% were female and 47% were male; 56.8% were single; most had university-level education or above (84.5%); most were unemployed or retired (54.9%); and most had a monthly income of <5000 Saudi Riyal (63%) (Table 2).

**Table 2:** Personal data of study participants, Western region, Saudi Arabia.

Personal data	#No	%
Age in yr		
18–29	227	61.7%
30–39	59	16.0%
40–49	34	9.2%
50+	48	13.0%
Gender		
Male	173	47.0%
Female	195	53.0%
Marital status		
Single	209	56.8%
Married	137	37.2%
Divorced/widow	22	6.0%
Education		
Below university	57	15.5%
University/above	311	84.5%
Occupation		
Unemployed/ retired	202	54.9%
Employed	166	45.1%
Monthly income		
<5000 SR	232	63.0%
5000–10,000 SR	55	14.9%
10,000–15,000 SR	47	12.8%
>15,000 SR	34	9.2%

SR = Saudi Riyal.

### 3.2. Collagen use among study participants

A total of 120 participants who have ever used collagen that represented 32.6% of all participants, most of them have used oral pharmaceutical form 64.2%, the results were similar in onset of collagen effect with slightly higher percentage in 2 months 26.7%. (Table 3). Nonusers were retained in the analysis because the study aimed to assess overall public knowledge and perceptions about collagen, not only user experiences.

**Table 3:** Collagen use among study participants, Western region, Saudi Arabia.

Collagen use	#No	%
Have you ever used collagen?		
Yes	120	32.6%
No	248	67.4%
What is the pharmaceutical form used? (n = 120)		
Oral	77	64.2%
Injection	8	6.7%
Topical	35	29.2%
If you used collagen before, when did you see its cosmetic results? (n = 120)		
1 month	31	25.8%
2 months	32	26.7%
3 months	28	23.3%
>3 months	29	24.2%

### 3.3. Public awareness and perception of collagen and its uses

According to the 1st section of the questionnaire that was on general knowledge of collagen, 72.8% of participants know that cosmetic uses of collagen and 64.1% knew that collagen is made naturally within body, whereas 84.8% did not know that there are 28 types of collagen, 3 of which are available in the Saudi market, and 72% did not knew that collagen production decrease in the body at the age of 18 years. According to the 2nd section on the factors and causes of collagen deficiency, 65.5% of participants did not knew that eating of sugar lead to decreased collagen production. In addition, 66.6% did not know that collagen production decreases by 1% each year after the age of 40 years, and the majority of participants (72%) did not know that vitamin C deficiency leads to decreased collagen synthesis. According to the 3rd section on the cosmetic effectiveness of collagen, 65.8% of participants knew that collagen requires months to show results, 81.5% knew the use of collagen helps decrease of wrinkles, and 76.1% of participants knew that collagen improves the growth and appearance of nails. According to the 4th section on the side effects, 58.2% of participants knew that collagen is considered to be a safe product that dispensed by pharmacists without prescriptions, but 70.4% of participants did not know that flatulence, dyspepsia, and nausea are among the few side effects resulting from collagen use (Table 4). Overall, awareness was mixed, with better understanding of cosmetic benefits but poor knowledge of biological mechanisms, deficiency factors, and side effects.

**Table 4:** Public awareness and perception regarding collagen and its uses in Western region, Saudi Arabia.

Domain	Knowledge/perception items	Yes		No/do not know	
		#No	%	#No	%
General knowledge	Do you know that collagen has multiple cosmetic uses?	68	72.8%	100	27.2%
	Do you know that collagen is made naturally in the body?	236	64.1%	132	35.9%
	Collagen is made of amino acids?	166	45.1%	202	54.9%
	Collagen contains 28 types, 3 types of them are available in the Saudi market?	56	15.2%	312	84.8%
	Do you have any knowledge about prices of collagen in Saudi markets?	140	38.0%	228	62.0%
	Collagen prices in Saudi markets are more expensive compared to international markets?	152	41.3%	216	58.7%
	Collagen production begins to decrease in the body at the age of 18?	103	28.0%	265	72.0%
	Collagen comes in many dosage forms, and it can be used orally, topically, or by injection?	220	59.8%	148	40.2%
	Pharmaceutical companies use marine and animal sources to manufacture collagen?	160	43.5%	208	56.5%
Factors and causes of collagen deficiency	Is smoking a cofactor in collagen deficiency?	161	43.8%	207	56.3%
	Frequent exposure to sunlight (ultraviolet) leads to collagen deficiency?	161	43.8%	207	56.3%
	Eating a lot of sugar contributes to decrease collagen production?	127	34.5%	241	65.5%
	Collagen production in the body decreases by 1% for each year after the age of 40?	123	33.4%	245	66.6%
	Scurvy (vitamin C deficiency) leads to a decrease in collagen synthesis?	103	28.0%	265	72.0%
Cosmetic effectiveness of collagen	Collagen needs a few months to show cosmetic results?	242	65.8%	126	34.2%
	Regular collagen use helps decreasing of wrinkles?	300	81.5%	68	18.5%
	Collagen contributes to the growth of nails and improve their appearance?	280	76.1%	88	23.9%
	Collagen can help the body by working as an antioxidant?	223	60.6%	145	39.4%
Side effects of collagen	Collagen is a safe cosmetic source, and is dispensed by a pharmacist without a prescription?	214	58.2%	154	41.8%
	Do you know that flatulence, dyspepsia, and nausea are among the few side effects associated with collagen use?	109	29.6%	259	70.4%

### 3.4. Distribution of participants’ overall knowledge levels regarding collagen and its use by their personal data

Of the participants between the ages of 18 and 29 years, 66.5% had poor knowledge of collagen. In contrast, 76.5% of participants between the ages of 40 and 49 years had good knowledge of collagen (*P*-value .001). Based on gender, 71.1% of male participants had poor knowledge of collagen compared to 49.2% of female participants (*P*-value .001). According to marital status, the majority (72.7%) of divorced or widowed participants had poor knowledge of collagen, while 42% of married participants and 69.4% of single participants had poor knowledge (*P*-value .001). Based on education level, 63.2% of those with a below-university level of education had poor knowledge of collagen, while 58.8% of participants with university-level education and above showed poor knowledge, with no statistical significance (*P*-value .542). In terms of occupation status, 64.4% of unemployed or retired participants had poor knowledge of collagen compared to 53.6% of employed participants (*P*-value .037). According to monthly income, 65.9% of participants whose monthly income was 5000 RS or less had poor knowledge of collagen (*P*-value .005) (Table 5). Effect sizes for significant associations indicated small to moderate effects. Participants with poor overall knowledge generally showed little awareness that collagen deficiency may affect physical function, such as causing muscle and joint weakness, reduced mobility, delayed wound healing, and functional difficulties that can contribute to disability.

**Table 5:** Distribution of participants overall knowledge level regarding collagen and its use by their personal data.

Personal data	Knowledge level				*P*-value
	Poor		Good		
	#No	%	#No	%	
Age in years					.001***^,^†
18–29	151	66.5%	76	33.5%	
30–39	32	54.2%	27	45.8%	
40–49	8	23.5%	26	76.5%	
50+	28	58.3%	20	41.7%	
Gender					.001***
Male	123	71.1%	50	28.9%	
Female	96	49.2%	99	50.8%	
Marital status					.001***^,^†
Single	145	69.4%	64	30.6%	
Married	58	42.3%	79	57.7%	
Divorced/widow	16	72.7%	6	27.3%	
Education					.542
Below university	36	63.2%	21	36.8%	
University/above	183	58.8%	128	41.2%	
Occupation					.037***
Unemployed/retired	130	64.4%	72	35.6%	
Employed	89	53.6%	77	46.4%	
Monthly income					.005***
<5000 SR	153	65.9%	79	34.1%	
5000–10,000 SR	25	45.5%	30	54.5%	
10,000–15,000 SR	21	44.7%	26	55.3%	
>15,000 SR	20	58.8%	14	41.2%	

*P* = Pearson *X*^2^ test, SR = Saudi Riyal.

† Exact probability test.

* *P* < .05 (significant).

** *P* < 0.01 (significant).

*** *P* < .001 (significant).

### 3.5. Distribution of participants’ knowledge levels regarding collagen and its use by history of collagen use

The general knowledge level of collagen users was good at 51.7%, compared to participants who had never used collagen (naïve) at 22.2% (*P*-value .001). Collagen users’ knowledge of the factors and causes of collagen deficiency was good at 31.7%, compared to naïve participants at 18.5% (*P*-value .005). Collagen users’ knowledge of the cosmetic effectiveness of collagen was good at 84.2%, compared to naïve participants at 60.5% (*P*-value .001). Collagen users’ knowledge about the side effects of collagen was poor at 69.2% and for naïve participants at 82.3% (*P*-value .004). However, collagen users’ overall knowledge was good at 65.8%, whereas it was 28.2% for naïve participants (*P*-value .001) (Table 6).

**Table 6:** Distribution of participants knowledge level regarding collagen and its use by history of using collagen.

Knowledge domains	Have you ever used collagen?				*P*-value
	Yes		No		
	No	%	No	%	
General knowledge level					.001***
Poor	58	48.3%	193	77.8%	
Good	62	51.7%	55	22.2%	
Factors and Causes of Collagen Deficiency					.005***
Poor	82	68.3%	202	81.5%	
Good	38	31.7%	46	18.5%	
Cosmetic effectiveness of Collagen					.001***
Poor	19	15.8%	98	39.5%	
Good	101	84.2%	150	60.5%	
Side effects of collagen					.004***
Poor	83	69.2%	204	82.3%	
Good	37	30.8%	44	17.7%	
Overall knowledge level					.001***
Poor	41	34.2%	178	71.8%	
Good	79	65.8%	70	28.2%	

*P* = Pearson *X*^2^ test.

† Exact probability test.

* *P* < .05 (significant).

** *P* < 0.01 (significant).

*** *P* < .001 (significant).

## 4. Discussion

In this study, we recruited a total of 368 participants. A study published in 2019 mentioned that collagen loss begins at 18 to 29 years of age, and then at the age of 40, collagen begins to be lost by about 1% yearly.^[15]^ In 1991, another study found that scurvy contributes to decrease collagen synthesis,^[10]^ and a study published in 2016 noted that exposure to UV radiation may decrease collagen production.^[13]^ In 2002, a study showed that smoking decreases collagen levels.^[15]^ In the current study, we hypothesized that the general public in the western region of Saudi Arabia will manifest limited knowledge about collagen use, and that their attitudes toward collagen consumption would be influenced by demographic factors such as age, gender, and cosmetic use habits. Findings showed that only 32.6% of participants had prior collagen use but knowledge on the same generally poor especially among younger adults and males. Participants with prior use of collagen demonstrated significantly higher knowledge compared to nonusers (65.8% vs 28.2%, *P* < .001), and awareness of side effects was low where about 78% of participants unaware of minor adverse effects. These findings imply limited knowledge, accordingly supporting the need for educational interventions to enhance safe and informed use of collagen products.

**Figure 1.**
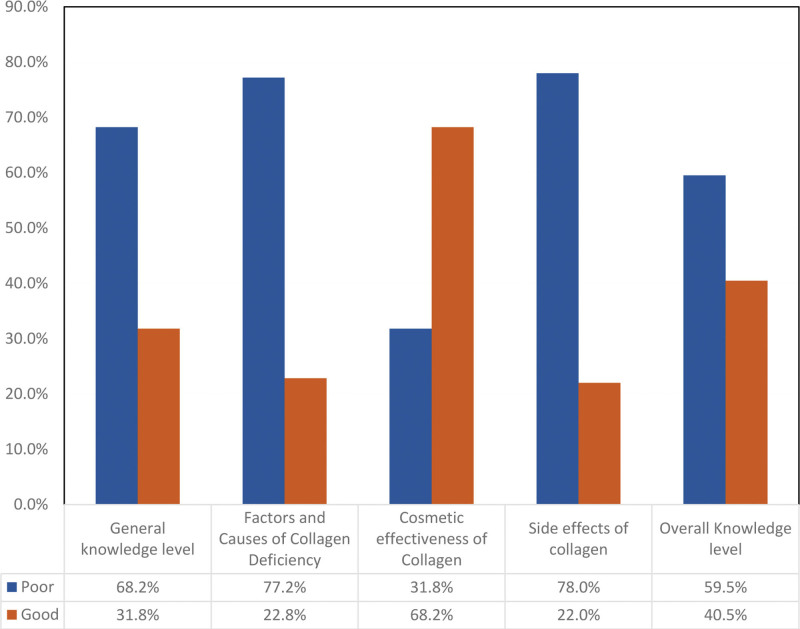
Knowledge and perception regarding collagen and its uses in the Western region of Saudi Arabia.

Regarding collagen effectiveness, randomized clinical studies published in 2014 and 2018 showed that collagen requires 1 to 3 months to exhibit its skin enhancing effects.^[27,28]^ In 2017, a randomized clinical trial suggested that collagen requires 24 weeks to show its effects for enhanced nail growth and appearance.^[29]^ Our results show that 68.2% of participants had good knowledge about the cosmetic effectiveness of collagen. This wide range of time required for collagen to show its effects may affect people’s adherence if they do not achieve the desired effect in a short duration (Fig. 1), particularly among younger adults who may expect faster results.

The World Health Organization and the European Commission for Health and Consumer Protection classify collagen as a safe cosmetic product with rare, minor side effects such as nausea, flatulence, or dyspepsia that could occur in some consumers.^[2]^ Our findings showed that 78% of participants had poor knowledge about collagen safety, which may lead to reluctance to use collagen. These knowledge gaps suggest that public misconceptions and misinformation could result in improper use, unrealistic expectations, or discontinuation of supplementation, highlighting the need for targeted education. Important to note in this dimension an as earlier indicated, collagen plays a critical role in maintaining the structural integrity and functional resilience of connective tissues, including cartilage, tendons, ligaments, and skin, all of which are essential for mobility and physical independence. Disruptions in collagen synthesis, organization, or degradation are implicated in a range of disabling conditions, such as osteoarthritis, musculoskeletal degeneration, fibrosis, and impaired wound healing. Further, age-related decline in collagen quality and turnover has the potential to worsen functional limitations thereby contributing to pain, stiffness, and reduced physical capacity. Consequently, educational strategies aimed at preserving collagen homeostasis or enhancing collagen regeneration are important necessary as they can provide meaningful implications for reducing disability and improving quality of life, particularly in aging and chronically ill populations.

Our study also found significant differences in knowledge levels according to age, gender, and prior collagen use. Younger participants (18–29 years) and males exhibited lower knowledge levels, potentially due to less exposure to educational resources or lower personal experience with collagen products. Collagen users, on the other hand, showed higher knowledge, indicating that personal experience contributes to awareness and understanding of its benefits.

### 4.1. Recommendations

Based on the findings of our study, we recommend that further studies be undertaken about knowledge of, perceptions of, and attitudes towards using collagen among people in other regions of Saudi Arabia. Based on the findings of other studies, we can develop awareness campaigns to educate people in various regions of Saudi Arabia about the effectiveness of collagen. In addition, collagen is considered to be a high-price product. We recommend that a pharmacoeconomic study examine the cost-effectiveness of collagen, which would help determine the price range of collagen products in Saudi Arabia.

### 4.2. Limitations of the study

First, according to our searching no study has investigated knowledge of, perceptions of, and attitudes towards using collagen among the population globally. Second, there is a lack of pharmacoeconomic studies discussing the price of collagen to help assess people’s attitudes towards the price of collagen products. Third, the restrictive COVID-19 protocols limited the number of survey responses obtained from elderly participants, who are less familiar with online survey format.

## 5. Conclusion

Collagen dysfunction represents a key biological pathway linking tissue degeneration to disability, highlighting its importance as a potential target for preventive and therapeutic interventions. The poor knowledge level manifested by participants in this study in a critical way can limit the benefits collagen uses among people. In previous research, collagen has been identified as a cosmetic product that elevates levels of collagen-derived peptides in the blood flow, leading to increases in skin properties such as elasticity, skin moisture, and skin firmness, and it also may decrease the progression of skin aging (dryness, laxity, and wrinkles) by its antioxidant effect. It has also been shown to affect the hair and nails and to protect the skin from UV light, and it is considered to be a safe product with tolerable side effects (gastrointestinal tract) according to a large number of studies. The significant effect of collagen for improving skin takes 1 to 3 months of consumption, in addition, it improves nails characteristics after 24 weeks. Accordingly, there is need for better educational interventions to help the wider population in Saudi Arabia attain the appropriate level of knowledge for safer and better usage of collagen products.

## Acknowledgments

The authors extend their appreciation to the King Salman center For Disability Research for funding this work through Research Group no KSRG-2024-227.

## Author contributions

**Conceptualization:** Ahmed M. Ashour, Nasser M. Alorfi, Mohammed M. Aldurdunji, Aliah Alhayyan.

**Data curation:** Ahmed M. Ashour, Mohammed M. Aldurdunji, Beisan A. Mohammad.

**Formal analysis:** Ahmed M. Ashour, Mohammed M. Aldurdunji, Beisan A. Mohammad, Naif H. Ashri, Ohood K. Almuzaini.

**Funding acquisition:** Ahmed M. Ashour, Nasser M. Alorfi, Fahad S. Alshehri.

**Investigation:** Ahmed M. Ashour, Fahad S. Alshehri, Beisan A. Mohammad, Naif H. Ashri, Ohood K. Almuzaini.

**Methodology:** Ahmed M. Ashour, Saad M. Wali, Nasser M. Alorfi, Bader A. Alhazmi, Mohammed M. Aldurdunji, Naif H. Ashri, Nasser M. Aldekhail.

**Project administration:** Ahmed M. Ashour, Bader A. Alhazmi, Mohammed M. Aldurdunji.

**Resources:** Ahmed M. Ashour, Fahad S. Alshehri, Mashael A. Alamri.

**Software:** Ahmed M. Ashour, Mohammed M. Aldurdunji, Nasser M. Aldekhail.

**Supervision:** Ahmed M. Ashour, Saad M. Wali, Mashael A. Alamri, Aliah Alhayyan.

**Validation:** Ahmed M. Ashour, Saad M. Wali, Fahad S. Alshehri, Bader A. Alhazmi.

**Visualization:** Ahmed M. Ashour, Aliah Alhayyan.

**Writing – original draft:** Ahmed M. Ashour.

**Writing – review & editing:** Ahmed M. Ashour, Bader A. Alhazmi.

## References

[1] Wang H. The potential of collagen treatment for comorbid diseases. Polymers (Basel). 2023;15:3999. 37836047 10.3390/polym15193999 PMC10574914

[2] Sibilla S Godfrey M Brewer S Budh-Raja A Genovese L. An overview of the beneficial effects of hydrolysed collagen as a nutraceutical on skin properties: scientific background and clinical studies. Tonutraj. 2015;8:29–42.

[3] Wu M Cronin K Crane JS. Biochemistry, Collagen Synthesis. StatPearls Publishing; 2020. 29939531

[4] Garnero P. The role of collagen organization on the properties of bone. Calcif Tissue Int. 2015;97:229–40. 25894071 10.1007/s00223-015-9996-2

[5] Salo A Myllyharju J. Prolyl and lysyl hydroxylases in collagen synthesis. Exp Dermatol. 2020;30:38–49. 32969070 10.1111/exd.14197

[6] Taguchi T Razzaque M. The collagen-specific molecular chaperone HSP47: is there a role in fibrosis? Trends Mol Med. 2007;13:45–53. 17169614 10.1016/j.molmed.2006.12.001

[7] Wang D Zhang M Guan H Wang X. Osteogenesis imperfecta due to combined heterozygous mutations in both COL1A1 and COL1A2, coexisting with pituitary stalk interruption syndrome. Front Endocrinol (Lausanne). 2019;10:193. 30984112 10.3389/fendo.2019.00193 PMC6447649

[8] Marini JC Reich A Smith SM. Osteogenesis imperfecta due to mutations in non-collagenous genes: lessons in the biology of bone formation. Curr Opin Pediatr. 2014;26:500–7. 25007323 10.1097/MOP.0000000000000117 PMC4183132

[9] Mao JR Bristow J. The Ehlers-Danlos syndrome: on beyond collagens. J Clin Invest. 2001;107:1063–9. 11342567 10.1172/JCI12881 PMC209288

[10] Peterkofsky B. Ascorbate requirement for hydroxylation and secretion of procollagen: relationship to inhibition of collagen synthesis in scurvy. Am J Clin Nutr. 1991;54:1135S–40S. 1720597 10.1093/ajcn/54.6.1135s

[11] Danessa G Notario D Regina R. Effects of collagen-based supplements on skin’s hydration and elasticity: a systematic review and meta-analysis. Indian J Dermatol Venereol Leprol. 2025;91:730–40. 40826844 10.25259/IJDVL_1165_2023

[12] Danby FW. Nutrition and aging skin: sugar and glycation. Clin Dermatol. 2010;28:409–11. 20620757 10.1016/j.clindermatol.2010.03.018

[13] Amano S. Characterization and mechanisms of photoageing-related changes in skin. Damages of basement membrane and dermal structures. Exp Dermatol. 2016;25:14–9. 27539897 10.1111/exd.13085

[14] Souyoul SA Saussy KP Lupo MP. Nutraceuticals: a review. Dermatol Ther (Heidelb). 2018;8:5–16. 29411317 10.1007/s13555-018-0221-x PMC5825326

[15] Knuutinen A Kokkonen N Risteli J. Smoking affects collagen synthesis and extracellular matrix turnover in human skin. Br J Dermatol. 2002;146:588–94. 11966688 10.1046/j.1365-2133.2002.04694.x

[16] León-López A Morales-Peñaloza A Martínez-Juárez VM Vargas-Torres A Zeugolis DI Aguirre-Álvarez G. Hydrolyzed collagen-sources and applications. Molecules. 2019;24:4031. 31703345 10.3390/molecules24224031 PMC6891674

[17] Shin JW Kwon SH Choi JY. Molecular mechanisms of dermal aging and antiaging approaches. Int J Mol Sci. 2019;20:2126. 31036793 10.3390/ijms20092126 PMC6540032

[18] Wang H. A review of the effects of collagen treatment in clinical studies. Polymers (Basel). 2021;13:3868. 34833168 10.3390/polym13223868 PMC8620403

[19] Pillai NS Khan AK Mehrotra N Jadhav KA. Comprehensive review on the role of collagen in health and disease. Biotech Res Asia. 2024;21:45–60.

[20] Sen CK Friday A Khanna S Roy S. Collagen-based products in wound, skin, and health care. Adv Wound Care (New Rochelle). 2025;14:21621918251361118. 10.1177/21621918251361118 PMC12359144 40720447

[21] Mathew-Steiner SS Roy S Sen CK. Collagen in wound healing. Bioengineering (Basel). 2021;8:63. 34064689 10.3390/bioengineering8050063 PMC8151502

[22] Charoenngam N Nasr A Shirvani A Holick MF. Hereditary metabolic bone diseases: a review of pathogenesis, diagnosis and management. Genes (Basel). 2022;13:1880. 36292765 10.3390/genes13101880 PMC9601711

[23] Dhillon RJ Hasni S. Pathogenesis and management of sarcopenia. Clin Geriatr Med. 2017;33:17–26. 27886695 10.1016/j.cger.2016.08.002 PMC5127276

[24] Campos LD Santos Junior VA Pimentel JD Carregã GLF Cazarin CBB. Collagen supplementation in skin and orthopedic diseases: a review of the literature. Heliyon. 2023;9:e14961. 37064452 10.1016/j.heliyon.2023.e14961 PMC10102402

[25] Pullar JM Carr AC Vissers MCM. The roles of vitamin C in skin health. Nutrients. 2017;9:866. 28805671 10.3390/nu9080866 PMC5579659

[26] Bolke L Schlippe G Gerß J Voss W. A collagen supplement improves skin hydration, elasticity, roughness, and density: results of a randomized, placebo-controlled, blind study. Nutrients. 2019;11:2494. 31627309 10.3390/nu11102494 PMC6835901

[27] Kim DU Chung HC Choi J Sakai Y Lee B-Y. Oral intake of low-molecular-weight collagen peptide improves hydration, elasticity, and wrinkling in human skin: a randomized, double-blind, placebo-controlled study. Nutrients. 2018;10:826. 29949889 10.3390/nu10070826 PMC6073484

[28] Proksch E Segger D Degwert J Schunck M Zague V Oesser S. Oral supplementation of specific collagen peptides has beneficial effects on human skin physiology: a double-blind, placebo-controlled study. Skin Pharmacol Physiol. 2014;27:47–55. 23949208 10.1159/000351376

[29] Hexsel D Zague V Schunck M Siega C Camozzato FO Oesser S. Oral supplementation with specific bioactive collagen peptides improves nail growth and reduces symptoms of brittle nails. J Cosmet Dermatol. 2017;16:520–6. 28786550 10.1111/jocd.12393

[30] Aguirre-Cruz G León-López A Cruz-Gómez V Jiménez-Alvarado R Aguirre-Álvarez G. Collagen hydrolysates for skin protection: oral administration and topical formulation. Antioxidants (Basel). 2020;9:181. 32098294 10.3390/antiox9020181 PMC7070905

[31] Tayyib N. Comparison of collagen wound dressings versus self-adaptive wound dressings in pressure ulcer treatment. J Wound Care. 2019;6:18–23.

[32] Genovese L Corbo A Sibilla S. An insight into the changes in skin texture and properties following dietary intervention with a nutricosmeceutical containing a blend of collagen bioactive peptides and antioxidants. Skin Pharmacol Physiol. 2017;30:146–58. 28528342 10.1159/000464470

[33] Schunck M Zague V Oesser S Proksch E. Dietary supplementation with specific collagen peptides has a body mass index-dependent beneficial effect on cellulite morphology. J Med Food. 2015;18:1340–8. 26561784 10.1089/jmf.2015.0022 PMC4685482

[34] Cosmetic Ingredient Review Expert Panel. Final report on the safety assessment of hydrolyzed collagen. J Am Coll Toxicol. 1985;4:199–221.

[35] Chiang TI Chang IC Lee HH. Amelioration of estrogen deficiency-induced obesity by collagen hydrolysate. Int J Med Sci. 2016;13:853–7. 27877077 10.7150/ijms.16706 PMC5118756

[36] Duarte GMF de Freitas KV Marini ACB. Acute supplementation with whey protein or collagen does not alter appetite in healthy women: a randomised double-blind and crossover pilot study. Br J Nutr. 2022;128:345–51. 34407895 10.1017/S0007114521003160

[37] Avila Rodríguez MI Rodríguez Barroso LG Sánchez ML. Collagen: a review on its sources and potential cosmetic applications. J Cosmet Dermatol. 2018;17:20–6. 29144022 10.1111/jocd.12450

[38] Rizk M Mostafa N. Extraction and characterization of collagen from buffalo skin for biomedical applications. Orient J Chem. 2016;32:1601–9.

[39] Tealab AA Maarouf AM Habous M Ralph DJ Abohashem S. The use of an acellular collagen matrix in penile augmentation: a pilot study in Saudi Arabia. Arab J Urol. 2013;11:169–73. 26558077 10.1016/j.aju.2013.02.001 PMC4442917

[40] Goresh HK Almarwani SH Alhomidani F. Assessing the significance and awareness of oral collagen in enhancing health and beauty among consumers in Saudi Arabia: a cross-sectional study. Cureus. 2025;17:e91149. 41018308 10.7759/cureus.91149 PMC12475036

[41] Ricard-Blum S. The collagen family. Cold Spring Harb Perspect Biol. 2011;3:a004978. 21421911 10.1101/cshperspect.a004978 PMC3003457

[42] Takashi T Mohammed SR. The collagen-specific molecular chaperone HSP47: is there a role in fibrosis. Trends Mol Med. 2007;13:45–53. 17169614 10.1016/j.molmed.2006.12.001

